# ﻿*Chlorocilliumsinense* sp. nov. (Clavicipitaceae) and *Calcarisporiumguizhouense* sp. nov. (Calcarisporiaceae) in Hypocreales from China

**DOI:** 10.3897/mycokeys.109.128060

**Published:** 2024-10-02

**Authors:** Wan-Hao Chen, Dan Li, Jian-Dong Liang, Xiu-Xiu Ren, Jie-Hong Zhao, Yan-Feng Han

**Affiliations:** 1 Center for Mycomedicine Research, Basic Medical School, Guizhou University of Traditional Chinese Medicine, Guiyang 550025, Guizhou, China; 2 Institute of Fungus Resources, Department of Ecology, College of Life Sciences, Guizhou University, Guiyang 550025, Guizhou, China; 3 Key Laboratory of Microbio and Infectious Disease Prevention & Control in Guizhou Province, Guiyang 550025, Guizhou, China; 4 College of Pharmacy, Guizhou University of Traditional Chinese Medicine, Guiyang 550025, Guizhou, China

**Keywords:** Entomopathogenic fungi, morphology, phylogenetic analysis, Sordariomycetes, taxonomic placement

## Abstract

Two new species, *Chlorocilliumsinense* and *Calcarisporiumguizhouense*, isolated from a spider and fruiting body of *Cordyceps* sp., are introduced. Morphological comparisons and phylogenetic analyses based on multigene datasets (ITS+LSU+*RPB2*+*tef*-1alpha) support the establishment of the new species. A combined dataset of ITS, LSU, *RPB2*, and *tef*-1alpha showed the taxonomic placement of *Chlorocillium* in Clavicipitaceae for the first time. *Pseudometarhizium* is regarded as a synonym of *Chlorocillium* and two *Pseudometarhizium* species are transferred into the latter based on the phylogenetic analysis and morphological characteristics.

## ﻿Introduction

During a survey of fungi associated with insects and spiders from Southwest China, a new spider-associated species and a new hyperparasitic species were found. The morphological characteristics and BLAST results revealed that the new collections belong to *Chlorocillium* and *Calcarisporium*. The genus *Chlorocillium* Zare & W. Gams was proposed to accommodate the species in VerticilliumsectionAlbo-erecta, and *Chlorocilliumgriseum* (Petch) Zare & W. Gams was described as the type species (Zare and Gams 2016). The typical characteristics of *Chlorocillium* are its slow-growing and greenish-ochraceous colony, conidiophores short with meagre whorls of phialides and fusiform conidia in chains (Zare and Gams 2016). The genus comprises the type species and two other species, *C.gueriniae* Y.P. Tan et al. and *C.montefioreae* Y.P. Tan et al. (Tan and Shivas 2023, 2024).

The genus *Calcarisporium* Preuss was introduced with *C.arbuscula* Preuss as the type species (Preuss 1851). The typical characteristics of *Calcarisporium* are its verticillate conidiophores, holoblastic conidiogenous cells, polyblastic, sympodial conidiation and ovoid to ellipsoidal conidia (Hughes 1951, de Hoog 1974). Sun et al. (2017) accepted six species i.e., *C.acerosum* Matsush., C. *arbuscula*, *C.cordycipiticola* Jing Z. Sun et al., *C.ovalisporum* (Petch) de Hoog, *C.phaeopodium* Somrith. & E.B.G. Jones and *C.xylariicola* Jing Z. Sun et al. Later, Zhu et al. (2023) introduced *C.yuanyangense* H. Yu bis & J.Y. Zhu.

In our phylogenetic analyses of combined ITS, LSU, *RPB2* and *tef1-α* sequences, *Chlorocillium* species clustered in Clavicipitaceae (Hypocreales, Hypocreomycetidae) with strong statistical support. Thus, we propose that *Chlorocillium* belongs to the family Clavicipitaceae. The new collections are phylogenetically and morphologically distinct from other known species. Thus, we introduce *Chlorocilliumsinese* sp. nov. and *Calcarisporiumguizhouense* sp. nov. Besides, *Pseudometarhizium* species groups with *Chlorocillium* s. str., and thus, we propose to synonymise *Pseudometarhizium* under *Chlorocillium* based on the phylogenetic analyses.

## ﻿Materials and methods

### ﻿Specimen collection and identification

The specimens were collected from Monkey-Ear Tiankeng (27°5'12.138"N, 107°0'48.42"E), Kaiyang County, Guiyang and Mayao River Valley (26°22'8.3748"N, 107°23'16.96"E), Duyun City, Qiannan Buyei and Miao Autonomous Prefecture, Guizhou Province, on 19 July 2023 and 1 May 2022. The samples were placed in an ice box and brought to the laboratory. Specimens were preserved in the refrigerator at 4 °C until further processing. The surface of each arthropod body was rinsed with sterile water, followed by sterilization with 75% ethanol for 3–5 s and rinsing again three times with sterilized water. After drying on sterilized filter paper, a piece of the synnemata, mycelium or sclerotia was cut from the specimen and placed on plates of potato dextrose agar (PDA) or PDA modified by the addition of 1% w/v peptone containing 0.1 g/l streptomycin and 0.05 g/l tetracycline (Chen et al. 2019). After fungal colonies emerged from the inoculated samples, a piece of mycelium from the colony edge was transferred onto new agar plates and cultured at 25 °C for 14 days under 12 h light/12 h dark conditions (Zou et al. 2010). The holotypes and ex-types were deposited at the Institute of Fungus Resources, Guizhou University (formally Herbarium of Guizhou Agricultural College; code, GZAC), Guiyang City, Guizhou, China. MycoBank numbers were obtained as outlined in MycoBank (http://www.MycoBank.org) (Crous et al. 2004).

Colony morphology was determined on PDA cultures incubated at 25 °C for 14 days and the growth rate, the presence of octahedral crystals and colony colors (surface and reverse) were observed. To investigate the microscopic characteristics, a little of the mycelia was picked up from the colony and mounted in lactophenol cotton blue or 20% lactic acid solution and the asexual morphological characteristics (e.g., conidiophores, phialides and conidia) were observed and measured using a Leica DM4 B microscope.

### ﻿DNA extraction, polymerase chain reaction amplification and nucleotide sequencing

DNA extraction was carried out using a fungal genomic DNA extraction kit (DP2033, BioTeke Corporation) according to Liang et al. (2011). The extracted DNA was stored at -20 °C. Polymerase chain reaction (PCR) was used to amplify genetic markers using the following primer pairs: ITS4/ITS5 for the internal transcribed spacer (ITS) region (White et al. 1990), LR0R/LR5 for 28s large subunit ribosomal (LSU) (Vilgalys and Hester 1990), fRPB2-5F/fRPB2-7cR for RNA polymerase II second largest subunit (*RPB2*) (Liu et al. 1999) and 983F/2218R for translation elongation factor 1 alpha (*tef*-1α) (Castlebury et al. 2004). The thermal cycle of PCR amplification for these phylogenetic markers was set up following the procedure described by Chen et al. (2021). PCR products were purified and sequenced at Sangon Biotech (Shanghai) Co. All newly generated sequences were deposited in GenBank and accession numbers were obtained (Table 1).

**Table 1.** List of strains and GenBank accession numbers of sequences used in this study.

Species	Strain No.	GenBank Accession No.			
		ITS	LSU	*RPB2*	*tef*–1α
*Aciculosporiumoplismeni*	MAFF 246966	LC571760	LC571760	LC572054	LC572040
*Aciculosporiumtake*	MAFF 241224	LC571753	LC571753	LC572048	LC572034
*Aciculosporiumtake*	TNS-F-60465	LC571755	LC571756	LC572049	LC572035
*Akanthomycesaculeatus*	HUA 772	KC519371	–	–	KC519366
*Aschersoniaconfluens*	BCC 7961	JN049841	DQ384947	DQ452465	DQ384976
*Aschersoniaplacenta*	BCC 7869	JN049842	EF469074	EF469104	EF469056
*Atkinsonellahypoxylon*	B4728	–	–	KP689514	KP689546
*Balansiaepichloe*	A.E.G. 96-15a	–	–	EF468908	EF468743
*Balansiahenningsiana*	A.E.G. 96-27a	JN049815	AY545727	DQ522413	AY489610
*Balansiapilulaeformis*	A.E.G. 94-2	–	AF543788	DQ522414	DQ522319
*Bionectriaochroleuca*	AFTOL-ID 187	–	DQ862027	DQ862013	DQ862029
*Bionectriavesiculosa*	HMAS 183151**^T^**	HM050304	HM050302	–	–
*Calcarisporiumarbuscula*	CBS 221.73	AY271809	–	–	–
*Calcarisporiumarbuscula*	CBS 900.68	KT945003	KX442598	KX442597	KX442596
*Calcarisporiumcordycipiticola*	CGMCC 3.17905	KT944999	KX442599	KX442594	KX442593
*Calcarisporiumcordycipiticola*	CGMCC 3.17904	KT945001	KX442604	KX442607	KX442605
***Calcarisporiumguizhouense***	**DY05041^T^**	**PP124948**	**PP133530**	–	**PP146564**
***Calcarisporiumguizhouense***	**DY05042**	**PP809658**	**PP809662**	–	**PP823899**
*Calcarisporiumxylariicola*	HMAS 276836**^T^**	KX442603	KX442601	KX442606	KX442595
*Calonectriailicicola*	CBS 190.50	GQ280605	GQ280727	KM232307	AY725726
*Cephalosporiumcurtipes*	CBS 154.61	AJ292404	AF339548	EF468947	EF468802
*Chlorocilliumgriseum*	CBS 387.73**^T^**	KU382150	KU382218	–	–
*Chlorocilliumgriseum*	RCEF6632	MW031768	MW084342	MW091329	MW091327
*Chlorocilliumgueriniae*	BRIP 72680a**^T^**	OR750699	OR731505	OR737788	OR737799
*Chlorocilliumgueriniae*	BRIP 72666a	OR750701	OR731507	OR737790	OR737801
*Chlorocilliumgueriniae*	BRIP 72668a	OR750702	OR731508	OR737791	OR737802
*Chlorocilliummontefioreae*	BRIP 70299a**^T^**	PP420202	PP415875	PP438395	PP438400
***Chlorocilliumsinense***	**KY07181^T^**	**PP768154**	**PP768156**	**PP766578**	**PP766580**
***Chlorocilliumsinense***	**KY07182**	**PP768155**	**PP768157**	**PP766579**	**PP766581**
*Clavicepsfusiformis*	ATCC 26019	JN049817	U17402	–	DQ522320
*Clavicepspurpurea*	GAM 12885	U57669	AF543789	DQ522417	AF543778
*Clavicepspurpurea*	S.A. cp11	–	EF469075	EF469105	EF469058
*Clonostachysrosea*	GJS90-227	–	AY489716	–	AY489611
*Collarinaaurantiaca*	FMR 11134	KJ807178	KJ807181	–	–
*Collarinaaurantiaca*	FMR 11784	KJ807177	KJ807180	–	–
*Conoideocrellaluteorostrata*	NHJ 11343	JN049859	EF468850	–	EF468801
*Conoideocrellaluteorostrata*	NHJ 12516	JN049860	EF468849	EF468946	EF468800
*Conoideocrellatenuis*	NHJ 6293	JN049862	EU369044	EU369087	EU369029
*Cocoonihabitussinensis*	HMAS254523**^T^**	KY924870	KY924869	–	–
*Cocoonihabitussinensis*	HMAS254524	MF687395	MF687396	–	–
*Corallocytostromaornithocopreoides*	WAC 8705	–	–	LT216620	LT216546
*Cordycepsbrongniartii*	BCC16585	JN049867	JF415967	JF415991	JF416009
*Cordycepsmilitaris*	OSC93623	JN049825	AY184966	–	DQ522332
*Dactylonectriaalcacerensis*	CBS 129087	JF735333	KM231629	–	JF735819
Dussiellatuberiformis*		–	–	JQ257020	JQ257027
*Elaphocordycepsophioglossoides*	NBRC 106332	JN943322	JN941409	–	–
*Elaphocordycepsparadoxa*	NBRC 106958	JN943324	JN941411	–	–
*Ephelisjaponica*	CBS 236.64	MH858427	–	–	–
*Ephelisjaponica*	Eph.oryzae	AB038564	–	–	–
*Ephelistripsaci*	CBS 857.72**^T^**	NR_153997	NG_059240	–	–
*Epichloeelymi*	C. Schardl 760	–	AY986924	–	AY986951
*Epichloetyphina*	ATCC 56429	JN049832	U17396	DQ522440	AF543777
*Flammocladiellaaceris*	CPC 24422	KR611883	KR611901	–	–
*Fusariumcircinatum*	CBS 405.97	U61677	–	JX171623	KM231943
*Fusariumsublunatum*	CBS 189.34**^T^**	HQ897830	KM231680	–	–
*Gelasinosporatetrasperma*	AFTOL-ID 1287	–	DQ470980	DQ470932	DQ471103
*Haptocilliumsinense*	CBS 567.95	AJ292417	AF339545	–	–
*Helicocollumsurathaniensis*	BCC 34463	–	KT222328	–	KT222336
*Helicocollumsurathaniensis*	BCC 34464**^T^**	–	KT222329	–	KT222337
*Heteroepichloebambusae*	Ba-01	AB065426	–	–	–
*Heteroepichloebambusae*	Bo-01	AB065428	–	–	–
*Heteroepichloesasae*	E.sasae-H	AB065432	–	–	–
*Heteroepichloesasae*	E.sasae-N	AB065431	–	–	–
*Hydropisphaeraerubescens*	ATCC 36093	–	AF193230	AY545731	DQ518174
*Hydropisphaeralutea*	ATCC 208838	–	AF543791	DQ522446	AF543781
*Hydropisphaerapeziza*	GJS92-101	–	AY489730	–	AY489625
*Hydropisphaerarufa*	DAOM JBT1003	JN942883	JN938865	–	–
*Hypocreaamericana*	AFTO -ID 52	DQ491488	AY544649	–	DQ471043
*Hypomycespolyporinus*	ATCC 76479	–	AF543793	–	AF543784
*Keithomycescarneus*	CBS 239.32	NR_131993	NG_057769	EF468938	EF468789
*Lecanicilliumattenuatum*	CBS 402.78	AJ292434	AF339565	EF468935	EF468782
*Lecanicilliumlecanii*	CBS 101247	JN049836	KM283794	KM283859	DQ522359
*Lecanicilliumpsalliotae*	CBS 367.86	–	KM283800	–	KM283823
*Marquandomycesmarquandii*	CBS 182.27	NR_131994	EF468845	EF468942	EF468793
*Marquandomyces* sp.	CBS 127132	MT078882	MT078857	MT078922	–
*Metapochoniabulbillosa*	CBS 145.70	MH859529	AF339542	EF468943	EF468796
*Metapochoniagonioides*	CBS 891.72	AJ292409	AF339550	DQ522458	DQ522354
*Metapochoniarubescens*	CBS 464.88**^T^**	–	AF339566	EF468944	EF468797
*Metapochoniasulchlasporia*	CBS 251.83	NR_154139	MH873311	–	KJ398790
*Metarhiziopsismicrospora*	CEHS133a	EF464589	EF464571	–	–
*Metarhiziopsismicrospora*	INEHS133a	EF464583	EF464572	–	–
*Metarhiziumanisopliae*	ARSEF 7487	HQ331446	–	DQ468370	DQ463996
*Metarhiziumanisopliae*	CBS 130.71**^T^**	MT078884	MT078853	MT078918	MT078845
*Metarhiziumflavoviride*	CBS 125.65	MT078885	MT078854	MT078919	MT078846
*Metarhiziumflavoviride*	CBS 700.74	–	MT078855	MT078920	MT078847
*Metarhiziumflavoviride*	CBS 218.56**^T^**	MH857590	MH869139	KJ398694	KJ398787
*Moelleriellaphyllogena*	CUP 067785	–	EU392610	–	EU392674
*Moelleriellaphyllogena*	CUP 067793	–	EU392608	–	EU392672
*Moelleriellaumbospora*	CUP 067817**^T^**	–	EU392628	–	EU392688
*Morakotiafusca*	BCC 64125	–	KY794862	–	KY794857
*Morakotiafusca*	BCC 79272**^T^**	–	KY794861	–	KY794856
*Mycophilomycespericoniae*	CPC 27558	NR_154209	NG_059746	–	–
*Myriogenosporaatramentosa*	A.E.G 96-32	–	AY489733	DQ522455	AY489628
*Myrotheciomycescorymbiae*	CPC 33206	NR_160351	NG_064542	–	–
*Myrotheciuminundatum*	IMI158855	–	AY489731	–	AY489626
*Myrotheciumroridum*	ATCC 16297	–	AY489708	–	AY489603
*Myrotheciumverrucaria*	ATCC 9095	–	AY489713	–	AY489608
*Nectriacinnabarina*	CBS 125165	HM484548	HM484562	KM232402	HM484527
*Nectrianigrescens*	CBS 125148	HM484707	HM484720	KM232403	HM484672
*Nectriopsisviolacea*	CBS 424.64	–	AY489719	–	–
*Neoaraneomycesaraneicola*	DY101711**^T^**	MW730520	MW730609	MW753026	MW753033
*Neoaraneomycesaraneicola*	DY101712	MW730522	MW730610	MW753027	MW753034
*Neobaryaparasitica*	Marson s/n	KP899626	KP899626	–	–
*Neonectriacandida*	CBS 151.29	JF735313	AY677333	–	JF735791
*Neonectriafaginata*	CBS 217.67	HQ840385	HQ840382	DQ789797	JF268746
*Neonectrianeomacrospora*	CBS 118984	HQ840388	HQ840379	DQ789810	JF268754
*Neonectriaramulariae*	CBS 182.36	HM054157	HM042435	DQ789793	HM054092
*Neurosporacrassa*	ICMP 6360	AY681193	AY681158	–	–
*Niessliaexilis*	CBS 560.74	–	AY489720	–	AY489614
*Nigeliaaurantiaca*	BCC13019	–	GU979948	GU979971	GU979957
*Nigeliamartiale*	EFCC 6863	–	JF415974	–	JF416016
*Ophiocordycepsheteropoda*	EFCC 10125	JN049852	EF468812	EF468914	EF468752
*Ophiocordycepssinensis*	EFCC 7287	JN049854	EF468827	EF468924	EF468767
*Ophiocordycepsstylophor*	OSC 111000	JN049828	DQ518766	DQ522433	DQ522337
*Orbiocrellapetchii*	NHJ 6209	JN049861	EU369039	EU369081	EU369023
*Orbiocrellapetchii*	NHJ 6240	–	EU369038	EU369082	EU369022
*Papiliomycesliangshanensis*	EFCC 1452	–	EF468815	–	EF468756
*Papiliomycesliangshanensis*	EFCC 1523	–	EF468814	EF468918	EF468755
*Papiliomycesshibinensis*	GZUH SB13050311**^T^**	NR154178	–	–	KR153589
*Parametarhiziumchangbaiense*	CGMCC 19143**^T^**	MN589741	MN589994	MT921829	MN908589
*Parametarhiziumhingganense*	CGMCC 19144	MN055703	MN061635	MT939494	MN065770
*Paraneoaraneomycessinensis*	ZY 22.006	OQ709254	OQ709260	OQ719621	OQ719626
*Paraneoaraneomycessinensis*	ZY 22.007	OQ709255	OQ709261	OQ719622	OQ719627
*Paraneoaraneomycessinensis*	ZY 22.008**^T^**	OQ709256	OQ709262	OQ719623	OQ719629
*Parepichloecinerea*	Ne-01	AB065425	–	–	–
*Peethambaraspirostriata*	CBS110115	–	AY489724	EF692516	AY489619
*Periglandulaipomoeae*	IasaF13	–	–	KP689517	KP689568
*Pochoniaboninensis*	JCM 18597	AB709858	AB709831	AB758693	AB758463
*Pochoniachlamydosporia*	CBS 101244	JN049821	DQ518758	DQ522424	DQ522327
*Pseudometarhiziumaraneogenum*	DY101741	MW730532	MW730618	MW753030	MW753037
*Pseudometarhiziumaraneogenum*	DY101742	MW730534	MW730619	MW753031	MW753038
*Pseudometarhiziumlepidopterorum*	SD05361**^T^**	MW730543	MW730624	–	MW753041
*Pseudometarhiziumlepidopterorum*	SD05362	MW730611	MW730629	–	MW753042
*Purpureomycesmaesotensis*	BCC 88441	MN781916	MN781877	MN781824	MN781734
*Purpureomycesmaesotensis*	BCC 85349	MN781928	MN781872	–	MN781729
*Purpureomycesmaesotensis*	BCC 89300**^T^**	MN781917	MN781876	–	MN781733
*Regiocrellacamerunensis*	ARSEF 7682	–	DQ118735	–	DQ118743
*Rosasphaeriamoravica*	LMM	JF440985	–	JF440986	JF440987
*Rotiferophthoraangustispora*	CBS 101437	AJ292412	AF339535	DQ522460	AF543776
*Roumegueriellarufula*	CBS 346.85	–	DQ518776	DQ522461	DQ522355
*Roumegueriellarufula*	GJS 91-164	–	EF469082	EF469116	EF469070
*Samuelsiachalalensis*	CUP 067856**^T^**	–	EU392637	–	EU392691
*Samuelsiamundiveteris*	BCC 40021	–	GU552152	–	GU552145
*Samuelsiarufobrunnea*	CUP 067858**^T^**	–	AY986918	–	AY986944
*Sarocladiumbacillisporum*	CBS 425.67	NR_145039	MH870718	–	–
*Sarocladiumdejongiae*	CBS 144929**^T^**	NR_161153	NG_067854	–	–
*Sarocladiumimplicatum*	CBS 959.72**^T^**	HG965023	MH878470	–	–
*Sarocladiumsubulatum*	CBS 217.35	MH855652	NG_070566	–	–
*Sarocladiumterricola*	CBS 243.59	MH857853	MH869389	–	–
*Shimizuomycesparadoxus*	EFCC 6279	JN049847	EF469084	EF469117	EF469071
*Shimizuomycesparadoxus*	EFCC 6564	–	EF469083	EF469118	EF469072
*Simplicilliumlamellicola*	CBS 116.25**^T^**	AJ292393	MH866307	DQ522462	DQ522356
*Simplicilliumlanosoniveum*	CBS 101267	AJ292395	–	DQ522463	DQ522357
*Simplicilliumlanosoniveum*	CBS 704.86	AJ292396	AF339553	DQ522464	DQ522358
*Sordariafimicola*	AFTOL-ID 216	DQ518178	–	–	DQ518175
*Stachybotryseucylindrospora*	ATCC 18851	JN942887	JN938869	–	–
*Sphaerostilbellaaureonitens*	GJS74-87	FJ442633	HM466683	FJ442763	–
*Sphaerostilbellaberkeleyana*	GJS82-274	–	U00756	–	AF543783
*Sphaerostilbellachlorohalonata*	DAOM 235557	JN942888	JN938870	–	–
*Stachybotrysmicrospora*	CBS 186.79	–	–	DQ676580	DQ676604
*Stephanonectriakeithii*	GJS92-133	–	AY489727	–	AY489622
*Sungiayongmunensis*	EFCC 2131**^T^**	JN049856	EF468833	–	EF468770
*Sungiayongmunensis*	EFCC 2135	–	EF468834	–	EF468769
*Tilachlidiumbrachiatum*	CBS 506.67	KM231839	HQ232177	KM232415	KM231976
*Tilachlidiumbrachiatum*	CBS 363.97	KM231838	KM231719	KM232414	KM231975
*Tolypocladiuminflatum*	SCALT1007-002	KC963032	–	–	–
*Trichodermaaggressivum*	CBS100525	–	JN939837	JQ014130	–
*Trichodermaviride*	GJS89-127	–	AY489726	–	AY489621
*Trichotheciumroseum*	DUCC 502	JN937590	JX458860	–	–
*Tyrannicordycepsfratricida*	TNS-F 19011	JQ349068	JQ257023	JQ257021	JQ257028
*Ustilaginoideadichromonae*	MRL IB9228	–	–	JQ257018	JQ257025
*Ustilaginoideavirens*	ATCC 16180	–	–	JQ257019	JQ257026
*Ustilaginoideavirens*	MAFF 240421	–	JQ257011	JQ257017	JQ257024
*Yosiokobayasiakusanagiensis*	TNS-F18494	–	JF415972	–	JF416014
*Pleurocordycepsaurantiaca*	MFLUCC 17-2113	MG136916	MG136910	MG136870	MG136875
*Pleurocordycepsmarginaliradians*	MFLU 17-1582**^T^**	MG136920	MG136914	MG271931	MG136878

Note: * J.F. White, Scale on Arundinaria tecta, North Carolina, 2000. New strains or species are in bold type.“^T^” denotes ex-type. Abbreviations for collections: ARSEF, USDA-ARS Collection of Entomopathogenic Fungal cultures, Ithaca, NY; ATCC, American Type Culture Collection, USA; BCC, BIOTEC Culture Collection, KlongLuang, Thailand; CBS, Centraalbureau voor Schimmelcultures, Utrecht, the Netherlands; CGMCC, China General Microbiological Culture Collection Center, China; EFCC, Entomopathogenic Fungal Culture Collection, Chuncheon, Korea; GZUH, Guizhou University Herbarium, Guiyang, Guizhou, China; HMAS, Herbarium of Mycology, Chinese Academy of Sciences; MAFF, Ministry of Agriculture, Forestry and Fisheries of Japan, Tokyo, Japan; NHJ, Nigel Hywel-Jones personal collection; OSC, Oregon State University Herbarium, Corvallis, OR; TNS-F, the mycological herbarium of the National Museum of Nature and Science, Tsukuba, Ibaraki, Japan; WAC, Western Australian Plant Pathology Reference Culture Collection, Australia, Perth.

### ﻿Sequence alignments and phylogenetic analyses

DNASTAR™ Lasergene (v 6.0) was used to edit DNA sequences in this study. The ITS, LSU, *RPB2* and *tef*-1α sequences were downloaded from GenBank, based on Sun et al. (2017), Chen et al. (2022a), Zhang et al. (2023), Zhu et al. (2023), and Tan and Shivas (2023, 2024) and others selected based on BLASTn searches in GenBank. ITS sequences and other loci were aligned and edited by MAFFT v.7.037b (Katoh and Standley 2013) and MEGA6 (Tamura et al. 2013). Combined sequences of ITS, LSU, *RPB2* and *tef*-1α were obtained using SequenceMatrix v.1.7.8 (Vaidya et al. 2011). The model was selected for Bayesian analysis by ModelFinder (Kalyaanamoorthy et al. 2017) in PhyloSuite (v1.2.2) software (Zhang et al. 2020).

The combined dataset of ITS, LSU, *RPB2* and *tef*-1α sequence data (analysis 1 and analysis 2) was analyzed using Bayesian inference (BI) and maximum likelihood (ML) methods. For BI, a Markov chain Monte Carlo (MCMC) algorithm was used to generate phylogenetic trees with Bayesian probabilities for the combined sequence datasets using MrBayes v.3.2 (Ronquist et al. 2012). The Bayesian analysis resulted in 20,001 trees after 10,000,000 generations. The first 4,000 trees, representing the burn-in phase of the analysis, were discarded, while the remaining 16,001 trees were used to calculate posterior probabilities in the majority rule consensus tree. After the analysis was finished, each run was examined using the program Tracer v.1.5 (Drummond and Rambaut 2007) to determine burn-in and confirm that both runs had converged. ML analyses were constructed with IQ-TREE (v 2.0) (Trifinopoulos et al. 2016), using an automatic selection of the model according to BIC.

## ﻿Results

### ﻿Phylogenetic analyses

**Figure 1.**
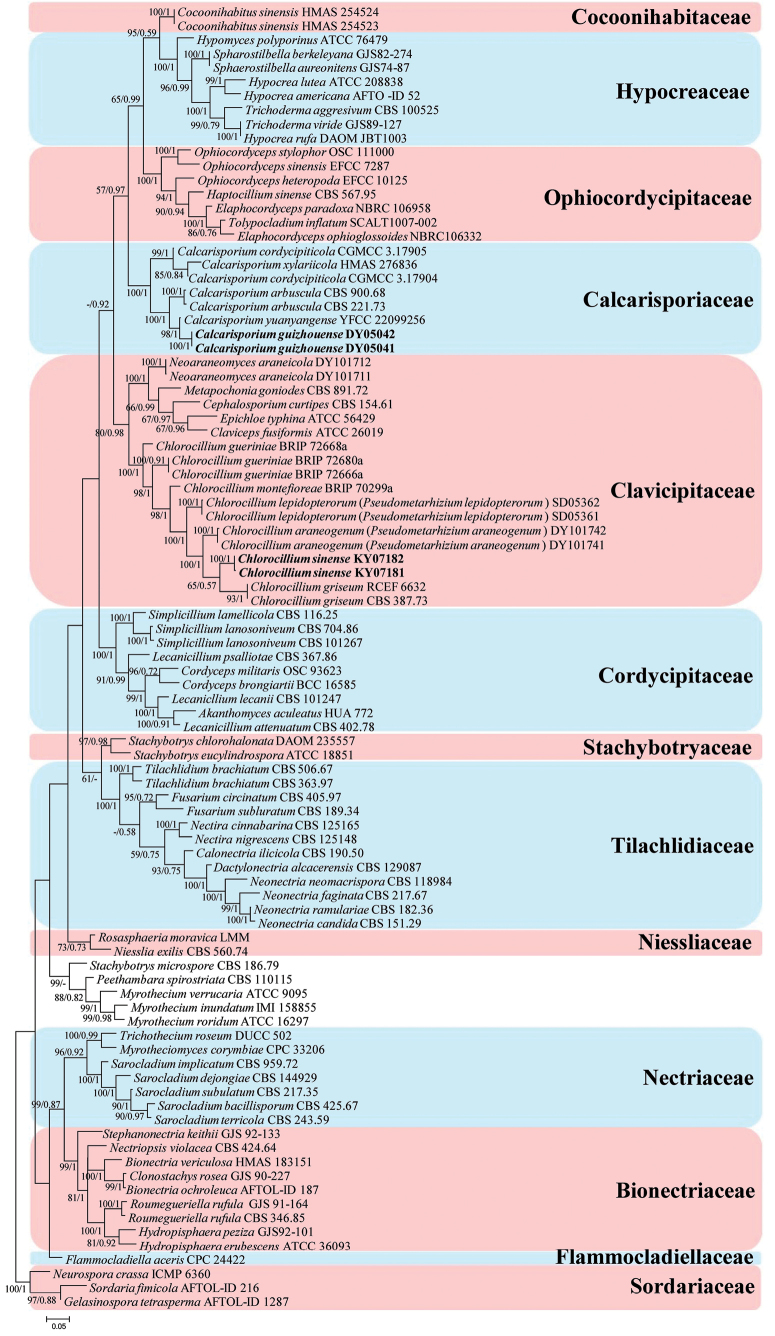
Phylogenetic analysis of the new strains and *Chlorocillium* species in the order Hypocreales based on multigene dataset (ITS, LSU, *RPB2* and *tef*-1α). Statistical support values (≥ 50%/0.5) are shown at the nodes for ML bootstrap support/BI posterior probabilities. The new taxa are in bold.

**Figure 2.**
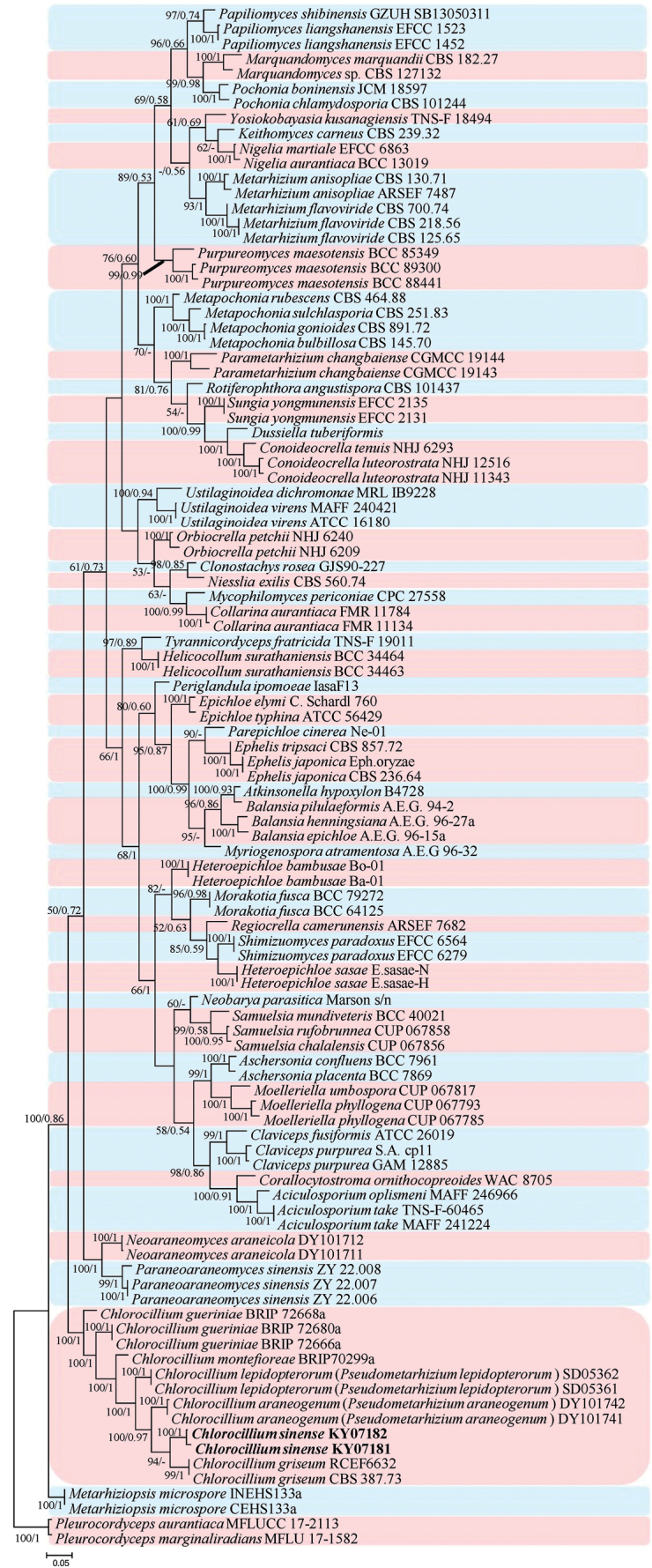
Phylogenetic analysis of *Chlorocillium* species in Clavicipitaceae, based on multigene dataset (ITS, LSU, *RPB2* and *tef*-1α). Statistical support values (≥ 50%/0.5) are shown at the nodes for ML bootstrap support/BI posterior probabilities. The new taxa are in bold.

The phylogenetic tree (Fig. 1) of analysis 1 was generated to determine the family placement of the new strains and *Chlorocillium* species. *Gelasinosporatetrasperma* Dowding (AFTOL-ID 1287), *Neurosporacrassa* Shear & B.O. Dodge (ICMP 6360) and *Sordariafimicola* (Roberge ex Desm.) Ces. & De Not. (AFTOL-ID 216) were used as the outgroup taxa in analysis 1. The concatenated sequences of analysis 1 included 79 taxa and consisted of 3,126 (ITS, 633; LSU, 842; *RPB2*, 846; and *tef*-1α, 805) characters with gaps. The phylogenetic tree (Fig. 2) of analysis 2 was generated to determine the establishment of *Chlorocillium* species in Clavicipitaceae (Suppl. material 1). *Pleurocordycepsaurantiaca* (Y.P. Xiao, T.C. Wen & K.D. Hyde) Y.H. Wang, et al. (MFLUCC 17-2113) and *Pleurocordycepsmarginaliradians* (Y.P. Xiao, T.C. Wen & K.D. Hyde) Y.H. Wang, et al. (MFLU 17-1582) were used as the outgroups in analysis 2. The concatenated sequences of analysis 2 included 69 taxa and consisted of 3,120 (ITS, 671; LSU, 740; *RPB2*, 825; and *tef*-1α, 884) characters with gaps.

Analysis 1: The selected model for ML analysis was TIM2+F+I+G4. The final value of the highest scoring tree was –46,827.254, which was obtained from an ML analysis of the dataset (ITS+LSU+*RPB2*+ *tef*-1α). The parameters of the rate heterogeneity model used to analyze the dataset were estimated using the following frequencies: A = 0.236, C = 0.274, G = 0.278, T = 0.211; substitution rates AC = 1.44290, AG = 2.23422, AT = 1.44290, CG = 1.00000, CT = 5.74279 and GT = 1.00000, as well as the gamma distribution shape parameter α = 0.630. The selected models for BI analysis were GTR+F+I+G4 (ITS, LSU and *RPB2*), and GTR+F+G4 (*tef*-1α). The phylogenetic trees (Fig. 1) constructed using ML and BI analyses were largely congruent and strongly supported in most branches. The new strains DY05041 and DY05042 clustered into a group of the family Calcarisporiaceae and have a close relationship with *Calcarisporiumarbuscula* (CBS 221.73 and CBS 900.68) and *C.yuanyangense* (YFCC 22099256). Strains KY07181, KY07182 and *Chlorocillium* species clustered as an independent clade and belong to the family Clavicipitaceae. Moreover, *Pseudometarhiziumaraneogenum* W.H. Chen, et al. (SD05361 and SD05362) and *P.lepidopterorum* W.H. Chen, et al. were grouped in *Chlorocillium* s. str. clade.

Analysis 2: The selected model for ML analysis was TN+F+I+G4. The final value of the highest scoring tree was –41,817.340, which was obtained from the ML analysis of the dataset (ITS+LSU+*RPB2*+ *tef*-1α). The parameters of the GTR model used to analyze the dataset were estimated based on the following frequencies: A = 0.233, C = 0.280, G = 0.277, T = 0.210; substitution rates AC = 1.00000, AG = 2.55486, AT = 1.00000, CG = 1.00000, CT = 5.56065 and GT = 1.00000, as well as the gamma distribution shape parameter α = 0.442. The selected models for BI analysis were GTR+F+I+G4 (ITS+LSU+ *tef*-1α) and SYM+G4 (*RPB2*). The phylogenetic trees (Fig. 2) constructed using ML and BI analyses were largely congruent and strongly supported in most branches. Most genera clustered into independent clades. Strains KY07181 and KY07182 clustered into an independent clade with close relationship with *Chlorocilliumgriseum*. However, *Pseudometarhizium* species clustered within the genus *Chlorocillium*.

### ﻿Taxonomy

#### ﻿ Calcarisporium guizhouense

Taxon classificationFungiHypocrealesCalcarisporiaceae

W.H. Chen, Y.F. Han & J.D. Liang sp. nov.

4DA59A3A-0FC6-5DE4-9709-A412B4BD9C87

854035

Fig. 3

##### Etymology.

Referring to its type location in Guizhou Province.

##### Type.

China • Guizhou Province, Qiannan Buyei and Miao Autonomous Prefecture, Duyun City, Mayao River Valley (26°22'8.3748"N, 107°23'16.96"E), on *Cordyceps* sp., 1 May 2022, Wanhao Chen, GZAC DY0504 (holotype), ex-type DY05041.

##### Description.

Colonies on PDA attaining a diameter of 28–29 mm after 14 days at 25 °C, white, consisting of a basal felt, floccose hyphal overgrowth, yellowish-white; reverse light brown to brown. Hyphae septate, hyaline, smooth-walled, 2.0–2.2 μm wide. Conidiophores erect, hyaline, verticillately branched, with 1–3 conidiogenous cells. Conidiogenous cells 15.6–23.2 × 1.5–1.7 μm, hyaline, cylindrical at base, gradually tapering near the apex, holoblastic to polyblastic, sympodial, apically with a cluster of conidium-bearing denticles. Conidia 5.2–8.6 × 1.8–2.2 μm, hyaline, smooth-walled, thin-walled, cylindrical, unicellular, acuminate.

**Figure 3.**
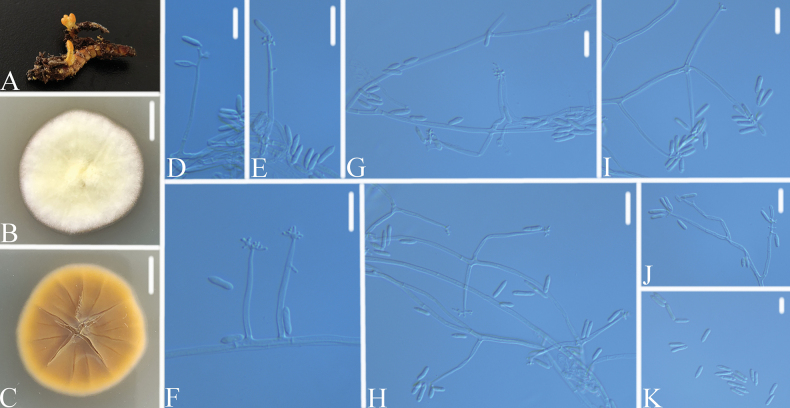
*Calcarisporiumguizhouense***A** the substrate *Cordyceps* sp. (fungicolous) **B**, **C**PDA culture plate showing top (**B**) and reverse (**C**) sides of the colony **D–K** Conidiogenous cells and conidia. Scale bars: 10 mm (**B**, **C**); 10 μm (**D–K**).

##### Substrate.

*Cordyceps* sp.

##### Additional strain examined.

China • Guizhou Province, Qiannan Buyei and Miao Autonomous Prefecture, Duyun City, Mayao River Valley (26°22'8.3748"N, 107°23'16.96"E). On *Cordyceps* sp., 1 May 2022, Wanhao Chen, DY05042 (living culture).

##### Notes.

*Calcarisporiumguizhouense* was easily identified as *Calcarisporium*, based on the BLASTn result in NCBI and its verticillate conidiophores and sympodial conidiation. Phylogenetic analyses show that *Calcarisporiumguizhouense* has close relationships to *C.arbuscula* and *C.yuanyangense* (Fig. 1). However, *C.guizhouense* was easily distinguished from *C.arbuscula* (Conidia: ovoid to ellipsoid, 4–11× 1.8–3.3 μm; substrate: decaying agaric) and *C.yuanyangense* (Conidia: ovoid to ellipsoid, 6.33–9.68 × 1.87–2.63 μm; substrate: *Ophiocordycepsnutans*) by its smaller cylindrical conidia and the substrate. Thus, the morphological characteristics and molecular phylogenetic results support *C.guizhouense* as a new species.

#### ﻿ Chlorocillium

Taxon classificationFungiHypocrealesCalcarisporiaceae

Zare & W. Gams, Mycol. Progr. 15: 1005, 2016

5CD9CBAE-D5A5-56DF-AC26-C23FC5995E59

= Pseudometarhizium W.H. Chen, Y.F. Han, J.D. Liang & Z.Q. Liang, MycoKeys 91: 59, 2022 MycoBank No: 842641.

##### Type species.

*Chlorocilliumgriseum* (Petch) Zare & W. Gams.

##### Notes.

Chen et al. (2022a) introduced the genus *Pseudometarhizium* with *P.araneogenum* (type species) and *P.lepidopterorum* in the family Clavicipitaceae. However, in the present study, the multi-gene phylogenetic analyses revealed that *Pseudometarhizium* species clustered with *Chlorocilliums. str.* (Fig. 2). Besides, the typical characteristics of *Pseudometarhizium* are entirely consistent with *Chlorocillium*. Thus, the genus *Pseudometarhizium* is synonymized under *Chlorocillium* and the two known species of *Pseudometarhizium* are transferred into *Chlorocillium*.

#### ﻿ Chlorocillium sinense

Taxon classificationFungiHypocrealesCalcarisporiaceae

W.H. Chen, Y.F. Han & J.D. Liang sp. nov.

AEADE5BA-7305-5834-951B-640B4D4B639F

853937

Fig. 4

##### Etymology.

Referring to the country where the fungus was first discovered.

##### Type.

China • Guizhou Province, Guiyang, Kaiyang County, Monkey-Ear Tiankeng (27°5'12.138"N, 107°0'48.42"E), on a dead spider (Araneae), 19 July 2023, Wanhao Chen, GZAC KY0718 (holotype), ex-type, KY07181.

##### Description.

Colonies on PDA reaching 15–17 mm in diameter in 14 days at 25 °C, green to yellowish green in center with white margin, reverse yellowish to light brown. Hyphae septate, hyaline, smooth-walled, 1.3–2.1 μm wide. Conidiophores hyaline, smooth-walled, emerging from aerial hyphae or chondroid mycelium, with single phialide or whorls of 2–4 phialides or verticillium-like from hyphae directly. Phialides cylindrical, somewhat inflated base, 11.7–20.1 × 1.1–1.3 μm, tapering to a thin neck. Conidia hyaline, smooth-walled, fusiform to ellipsoidal, 1.9–2.9 × 0.8–1.2 μm, forming divergent and basipetal chains. Octahedral crystals and chlamydospores absent.

**Figure 4.**
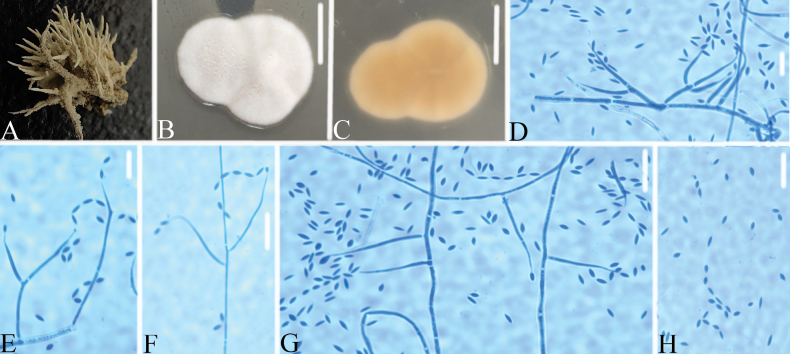
*Chlorocilliumsinense***A** infected spider **B, C**PDA-containing culture plate showing **B** the front and **C** reverse sides of the colony **D–H** phialides, conidia in chains and conidia. Scale bars: 10mm (**B**, **C**); 10 μm (**D**–**H**).

##### Host.

Spider (Araneae).

##### Additional strain examined.

China • Guizhou Province, Guiyang, Kaiyang County, Monkey-Ear Tiankeng (27°5'12.138"N, 107°0'48.42"E). On a dead spider (Araneae), 19 July 2023, Wanhao Chen, KY07182 (living culture).

##### Remarks.

*Chlorocilliumsinense* was easily identified as *Chlorocillium*, based on the BLASTn result in NCBI. The phylogenetic analysis of the combined dataset of ITS, LSU, *RPB2* and *tef*-1α sequence data showed that the new collections clustered as an independent clade with close relationship to *C.araneogenum*, *C.griseum* and *C.lepidopterorum* (Fig. 2). Table 2 provides the different morphological characteristics that can be used to differentiate *Chlorocilliumsinense* from other phylogenetically close species.

**Table 2.** Morphological comparison of the new species with other *Chlorocillium* species.

Species	Strain	Phialides (μm)	Conidia (μm)	Host	Octahedral crystals
*C.araneogenum*	DY101801	8.3–23.3 × 1.3–2.2	fusiform, 3.4–5.8 × 1.4–1.8	spider	absent
*C.griseum*	CBS 387.73	18–40(−55) × 2–2.5	fusiform, 4.5–6 × 1.0–1.5	spider	present
*C.lepidopterorum*	SD05361	21.2–33.7 × 1.1–1.4	fusiform, 3.1–4.3 × 1.3–1.5	pupa	absent
*C.sinense*	KY07181	11.7–20.1 × 1.1–1.3	fusiform to ellipsoidal, 1.9–2.9 × 0.8–1.2	spider	absent

### ﻿New combinations

#### ﻿ Chlorocillium araneogenum

Taxon classificationFungiHypocrealesCalcarisporiaceae

(W.H. Chen, Y.F. Han, J.D. Liang & Z.Q. Liang) W.H. Chen, Y.F. Han & J.D. Liang comb. nov.

479E2C58-94FD-5E73-93C2-482E8E5B61A0

853934

Pseudometarhizium araneogenum W.H. Chen, Y.F. Han, J.D. Liang & Z.Q. Liang, MycoKeys 91: 60 (2022) (Basionym).

##### Description.

Colonies irregular on PDA, 1.8–2.8 cm diam. after 14 d at 25 °C, white, consisting of a basal felt, floccose hyphal overgrowth, reverse yellowish to pale brown or green. Prostrate hyphae smooth, septate, hyaline, 1.0–1.2 μm diam. Erect conidiophores usually arising from aerial hyphae. Phialides solitary or in groups of two, 8.3–23.3 × 1.3–2.2 μm, with a cylindrical basal portion, tapering into a short distinct neck. Conidia in chains, hyaline, fusiform, one-celled, 3.4–5.8 × 1.4–1.8 μm.

##### Material examined (type material).

China • Duyun City, Qiannan Buyi and Miao Autonomous Prefecture, Guizhou Province. On a dead spider (Araneae), 1 October 2019, Wanhao Chen, GZAC DY10174, living cultures, DY101741, DY101742.

#### ﻿ Chlorocillium lepidopterorum

Taxon classificationFungiHypocrealesCalcarisporiaceae

(W.H. Chen, Y.F. Han, J.D. Liang & Z.Q. Liang) W.H. Chen, Y.F. Han & J.D. Liang comb. nov.

37C79B02-B1AE-5142-9678-31D5D2BE83CC

853935

Pseudometarhizium lepidopterorum W.H. Chen, Y.F. Han, J.D. Liang & Z.Q. Liang, MycoKeys 91: 60 (2022) (Basionym).

##### Description.

Colonies on PDA, 1.4–2.0 cm diam. after 14 d at 25 °C, white, consisting of a basal felt and cottony, floccose hyphal overgrowth, reverse yellowish to pale green. Prostrate hyphae smooth, septate, hyaline, 1.0–2.0 μm diam. Erect conidiophores usually arising from aerial hyphae. Phialides solitary or in groups of two to three, 21.2–33.7 × 1.1–1.4 μm, with a cylindrical basal portion, tapering into a short distinct neck. Conidia in chains, hyaline, fusiform, one-celled, 3.1–4.3 × 1.3–1.5 μm.

##### Material examined (type material).

China • Sandu County, Qiannan Buyi and Miao Autonomous Prefecture, Guizhou Province, . On a pupa (Lepidoptera), 1 May 2019, Wanhao Chen, GZAC SD0536, living cultures, SD05361, SD05362.

## ﻿Discussion

The complex terrain, mild climate, abundant rainfall, wide vegetation coverage, and diverse forest types and components, result in Guizhou Province having abundant resources of fungi associated with insects. However, while fungal species associated with insects are often found in forest and grassland reservations (Chen et al. 2019, 2020, Peng et al. 2023, Xiao et al. 2023, 2024, Liu et al. 2024); they are rarely found in special karst eco-environments, such as Tiankeng and valleys. Chen et al. (2022a, b, 2023) introduced thirteen new species associated with insects or spiders from Tiankeng or valleys.

*Calcarisporium* species are often reported as fungicolous and produce abundant secondary metabolites (Sun et al. 2017, Zhu et al. 2023). Three species, *C.cordycipiticola*, *C.ovalisporum* and *C.yuanyangense* were introduced from the entomopathogenic taxa, *Cordycepsmilitaris* (L.) Fr., *Hirsutellacitriformis* Speare and *Ophiocordycepsnutans* (Pat.) G.H. Sung et al. In the present study, *Calcarisporiumguizhouense* were introduced with the substrate *Cordyceps* sp. from Mayao River Valley.

Zare and Gams (2016) introduced the genus *Chlorocillium* with *C.griseum*, and the placement of the genus was not confirmed in Hypocreomycetidae. Wang (2022) noted that the genus *Chlorocillium* belongs to the order Hypocreales and has a close relationship with the family Clavicipitaceae. Tan and Shivas (2023, 2024) reported two new *Chlorocillium* species in Australia. However, the taxonomic status of the genus was still unclear.

In the order-level phylogenetic tree (Fig. 2), *Chlorocillium* species clustered into Clavicipitaceae (Hypocreales, Hypocreomycetidae, Sordariomycetes). Thus, the combined dataset of ITS, LSU, *RPB2*, and *tef*-1α showed the taxonomic placement of *Chlorocillium* in Clavicipitaceae for the first time. Furthermore, in the family-level phylogenetic tree (Fig. 1), strains KY07181 and KY07182 formed an independent branch and clustered with *C.griseum*, *C.gueriniae*, *C.montefioreae*, *P.araneogenum* and *P.lepidopterorum* in a subclade. *Pseudometarhizium* species were also consistent with the genus *Chlorocillium* based on the typical characteristics. Thus, we synonymized *Pseudometarhizium* under *Chlorocillium* and transferred its species.

## ﻿Conclusion

Two new species, *Chlorocilliumsinense* and *Calcarisporiumguizhouense*, were established and described in the present study. The genus *Chlorocillium* was confirmed in the family Clavicipitaceae. Furthermore, the genus *Pseudometarhizium* was synonymized with *Chlorocillium* and its species were transferred.

## Supplementary Material

XML Treatment for Calcarisporium guizhouense

XML Treatment for Chlorocillium

XML Treatment for Chlorocillium sinense

XML Treatment for Chlorocillium araneogenum

XML Treatment for Chlorocillium lepidopterorum

## ﻿Additional information

### Author ORCIDs

Wan-Hao Chen https://orcid.org/0000-0001-7240-6841

Jian-Dong Liang https://orcid.org/0000-0002-3939-3900

Yan-Feng Han https://orcid.org/0000-0002-8646-3975

### Data availability

All of the data that support the findings of this study are available in the main text or Supplementary Information.

## Supplementary materials

Supplementary material 1 The genus contain in the family Clavicipitaceae Data type docx https://binary.pensoft.net/file/1146896This dataset is made available under the Open Database License (http://opendatacommons.org/licenses/odbl/1.0/). The Open Database License (ODbL) is a license agreement intended to allow users to freely share, modify, and use this Dataset while maintaining this same freedom for others, provided that the original source and author(s) are credited.Wan-Hao Chen, Dan Li, Jian-Dong Liang, Xiu-Xiu Ren, Jie-Hong Zhao, Yan-Feng Han
