# Supplementary material for: ﻿Chlorocilliumsinense sp. nov. (Clavicipitaceae) and Calcarisporiumguizhouense sp. nov. (Calcarisporiaceae) in Hypocreales from China
Source: MycoKeys. 2024 Oct 2;109:91–107. doi: 10.3897/mycokeys.109.128060 (PMC11464903; doi:10.3897/mycokeys.109.128060)
Supplement: Supplementary material 1 — The genus contain in the family Clavicipitaceae [file mycokeys-109-091-s001.docx]

Table S1. The genus contain in the family Clavicipitaceae

| Number | Genus | Type species | DNA sequences |
| --- | --- | --- | --- |
| 1 | *Aciculosporium* | *Aciculosporium take* | Available |
| 2 | *Aschersonia* | *Aschersonia tahitensis* | Available |
| 3 | *Atkinsonella* | *Atkinsonella hypoxylon* | Available |
| 4 | *Balansia* | *Balansia claviceps* | Available |
| 5 | *Cavimalum* | *Cavimalum indicum* | Absent |
| 6 | *Claviceps* | *Claviceps purpurea* | Available |
| 7 | *Collarina* | *Collarina aurantiaca* | Available |
| 8 | *Conoideocrella* | *Conoideocrella luteorostrata* | Available |
| 9 | *Corallocytostroma* | *Corallocytostroma oryzae* | Available |
| 10 | *Dussiella* | *Dussiella tuberiformis* | Available |
| 11 | *Ephelis* | *Ephelis mexicana* | Available |
| 12 | *Epichloe* | *Epichloe typhina* | Available |
| 13 | *Epicrea* | *Epicrea insignis* | Absent |
| 14 | *Helicocollum* | *Helicocollum surathaniense* | Available |
| 15 | *Helminthascus* | *Helminthascus arachnophthorus* | Absent |
| 16 | *Heteroepichloe* | *Heteroepichloe bambusae* | Available |
| 17 | *Keithomyces* | *Keithomyces carneus* | Available |
| 18 | *Konradia* | *Konradia bambusina* | Absent |
| 19 | *Loculistroma* | *Loculistroma bambusae* | Absent |
| 20 | *Marquandomyces* | *Marquandomyces marquandii* | Available |
| 21 | *Metapochonia* | *Metapochonia suchlasporia* | Available |
| 22 | *Metarhiziopsis* | *Metarhiziopsis microspora* | Available |
| 23 | *Metarhizium* | *Metarhizium anisopliae* | Available |
| 24 | *Moelleriella* | *Moelleriella sulphurea* | Available |
| 25 | *Mycomalus* | *Mycomalus bambusinus* | Absent |
| 26 | *Mycophilomyces* | *Mycophilomyces periconiae* | Available |
| 27 | *Myriogenospora* | *Myriogenospora paspali* | Available |
| 28 | *Neobarya* | *Neobarya parasitica* | Available |
| 29 | *Neocordyceps* | *Neocordyceps kohyasanensis* | Absent |
| 30 | *Nigelia* | *Nigelia aurantiaca* | Available |
| 31 | *Nigrocornus* | *Nigrocornus scleroticus* | Absent |
| 32 | *Orbiocrella* | *Orbiocrella petchii* | Available |
| 33 | *Papiliomyces* | *Papiliomyces liangshanensis* | Available |
| 34 | *Parametarhizium* | *Parametarhizium hingganense* | Available |
| 35 | *Paraneoaraneomyces* | *Paraneoaraneomyces sinensis* | Absent |
| 36 | *Parepichloe* | *Parepichloe cinerea* | Available |
| 37 | *Periglandula* | *Periglandula ipomoeae* | Available |
| 38 | *Pochonia* | *Pochonia humicola* | Available |
| 39 | *Pseudomeria* | *Pseudomeria mucosa* | Absent |
| 40 | *Purpureomyces* | *Purpureomyces khaoyaiensis* | Available |
| 41 | *Regiocrella* | *Regiocrella camerunensis* | Available |
| 42 | *Romanoa* | *Romanoa terricola* | Available |
| 43 | *Rotiferophthora* | *Rotiferophthora globospora* | Available |
| 44 | *Samuelsia* | *Samuelsia rufobrunnea* | Available |
| 45 | *Shimizuomyces* | *Shimizuomyces paradoxus* | Available |
| 46 | *Sphaerocordyceps* | *Sphaerocordyceps palustris* | Absent |
| 47 | *Sungia* | *Sungia yongmunensis* | Available |
| 48 | *Tyrannicordyceps* | *Tyrannicordyceps fratricida* | Available |
| 49 | *Ustilaginoidea* | *Ustilaginoidea oryzae* | Available |
| 50 | *Yosiokobayasia* | *Yosiokobayasia kusanagiensis* | Available |
